# Supplementary material for: Metabolic disorders and the risk of head and neck cancer: a protocol for a systematic review and meta-analysis
Source: BMJ Open. 2022 May 4;12(5):e058392. doi: 10.1136/bmjopen-2021-058392 (PMC9073393; doi:10.1136/bmjopen-2021-058392)
Supplement: Author's manuscript [file bmjopen-2021-058392.draft_revisions.pdf]

BMJ Open is committed to open peer review. As part of this commitment we make the peer review history of every article we publish publicly available.

When an article is published we post the peer reviewers' comments and the authors' responses online. We also post the versions of the paper that were used during peer review. These are the versions that the peer review comments apply to.

The versions of the paper that follow are the versions that were submitted during the peer review process. They are not the versions of record or the final published versions. They should not be cited or distributed as the published version of this manuscript.

BMJ Open is an open access journal and the full, final, typeset and author-corrected version of record of the manuscript is available on our site with no access controls, subscription charges or pay-per-view fees (http://bmjopen.bmj.com).

If you have any questions on BMJ Open's open peer review process please email info.bmjopen@bmj.com

BMJ Open

Metabolic disorders and the risk of head and neck cancer: a protocol for a systematic review and meta-analysis

Journal:	*BMJ Open*
Manuscript ID	bmjopen-2021-058392
Article Type:	Protocol
Date Submitted by the Author:	18-Oct-2021

Complete List of Authors:	Gormley, Alexander; University of Bristol, Dental School, Faculty of Health Sciences; Bristol Dental Hospital, University Hospitals Bristol and Weston NHS Foundation Trust<br>Richards, Charlotte; Cardiff University, School of Dentistry<br>Spiga, Francesca; University of Bristol, Population Health Sciences, Bristol Medical School, Faculty of Health Sciences; University of Bristol, MRC Integrative Epidemiology Unit, Population Health Sciences, Bristol Medical School, Faculty of Health Sciences<br>Gray, Emily; University Hospitals Bristol and Weston NHS Foundation Trust<br>Hooper, Joanna; Library and information services, University Hospitals Bristol and Weston NHS Foundation Trust<br>Main, Barry; University of Bristol, Bristol Dental School, Faculty of Health Sciences, University of Bristol; University of Bristol, Population Health Sciences, Faculty of Health Sciences<br>Vincent, Emma; University of Bristol, MRC Integrative Epidemiology Unit, Population Health Sciences, Bristol Medical School, Faculty of Health Sciences; University of Bristol, School of Cellular and Molecular Medicine, Faculty of Life Sciences<br>Richmond, Rebecca; University of Bristol, Population Health Sciences, Bristol Medical School, Faculty of Health Sciences; University of Bristol, MRC Integrative Epidemiology Unit, Population Health Sciences, Bristol Medical School, Faculty of Health Sciences<br>Higgins, Julian; University of Bristol, Population Health Sciences, Bristol Medical School, Faculty of Health Sciences; University of Bristol, MRC Integrative Epidemiology Unit, Population Health Sciences, Bristol Medical School, Faculty of Health Sciences<br>Gormley, Mark; University of Bristol, Bristol Dental School, Faculty of Health Sciences; University of Bristol, MRC Integrative Epidemiology Unit, Population Health Sciences, Bristol Medical School, Faculty of Health Sciences

Keywords:	ONCOLOGY, ORAL & MAXILLOFACIAL SURGERY, Head & neck tumours < ONCOLOGY

Metabolic disorders and the risk of head and neck cancer: a protocol for a systematic review and meta-analysis

Alexander Gormley[1,2], Charlotte Richards[3*], Francesca Spiga[4,5], Emily Gray[2], Joanna Hooper[6], Barry G Main[1,2,4], Emma E Vincent[4,5,7], Rebecca C Richmond[4,5], Julian PT Higgins[4,5], Mark Gormley[1,2,4,5]*

1 – Bristol Dental School, Faculty of Health Sciences, University of Bristol

2 – Bristol Dental Hospital, University Hospitals Bristol and Weston NHS Foundation Trust

3 – School of Dentistry, Cardiff University

4 – Population Health Sciences, Bristol Medical School, Faculty of Health Sciences, University of Bristol

5 – MRC Integrative Epidemiology Unit, Population Health Sciences, Bristol Medical School, Faculty of Health Sciences, University of Bristol

6 – Library and information services, University Hospitals Bristol and Weston NHS Foundation Trust

7 – School of Cellular and Molecular Medicine, Faculty of Life Sciences, University of Bristol

* - Joint first authors

Corresponding author – mark.gormley@bristol.ac.uk

Mark Gormley

Bristol Dental School

University of Bristol

Lower Maudlin Street

Bristol

BS1 2LY

Abstract

Introduction

Head and neck cancer squamous cell carcinoma (HNSCC) is the 6th most common cancer internationally. Established risk factors include smoking, alcohol and presence of human papillomavirus (HPV). The incidence rate of new disease continues to rise, despite falls in alcohol consumption and a reduction in smoking, the rising rates are unlikely to be solely attributed to HPV status alone. Obesity and its associated conditions such as type 2 diabetes (T2D) are implicated in the risk and progression of a variety of cancers but there is paucity of evidence regarding its role in HNSCC.

Methods and analysis

A systematic review of cohort studies, reporting a risk of incident head and neck squamous cell carcinoma will be included. A systematic search strategy has been developed, multiple databases will be searched from January 1966, including Cochrane Library, OVID SP versions of Medline and EMBASE. The primary outcome will be incident HNSCC based on exposures of type 2 diabetes, obesity, dyslipidemia and hypertension as defined by the World Health Organisation (WHO). A combined risk effect across studies will be calculated using meta-analysis, although depending on the heterogeneity in study design, exposure and outcome reporting this may not be possible.

Ethics and dissemination

No ethical approval is required for this systematic review. The review will be published in a revelant peer-review journal and findings will be presented at scientific meetings in both poster and oral presentation form.

Registration details

This study has been registered with the international prospective register of systematic reviews (PROSPERO) with study registration number CRD42021250520. This protocol has been developed in accordance with the preferred reporting items for systematic review and meta-analysis protocols (PRISMA-P) guidance statement.

Article Summary

Strengths and limitations of this study

- *This systematic review will be the first to comprehensively review the literature and provide a meta-analysis of the effect of metabolic disorders and the risk of incident HNSCC.*
- *The publication of this protocol provides a clear representation of the methods used in this review for transparency and to prevent future duplication.*
- *This systematic review will be one of the first to pilot the The ROBINS-E tool (Risk Of Bias In Non-randomised Studies - of Exposures).*
- *Despite the metabolic disorders being described as separate entities, the authors recognise the potential for disease processes to be related.*
- *Among cohort studies there is significant variability in terms of length of follow-up for the outcome of interest, HNSCC.*

Introduction

Head and neck squamous cell carcinoma (HNSCC), which includes cancer of the oral cavity, oropharynx and larynx, is the world's 6th most common cancer, with the highest incidence in males and those over 70 years old.[1] Established risk factors include smoking, alcohol and the human papillomavirus (HPV), which has mainly been linked to oropharyngeal cancer.[2] Despite the significant reduction in smoking,[3] and a fall in alcohol consumption,[4] in the UK over the previous two decades, incidence rates of head and neck cancers have continued to rise by around a third.[1] Given this potentially changing aetiology,[2] further exploration of less well known risk factors is warranted to target prevention and identify patients with early disease. People living with obesity and associated conditions such as type 2 diabetes (T2D), have an increased risk of developing certain cancers (e.g. liver and pancreas), but the epidemiological evidence surrounding HNSCC is not conclusive.[5, 6] Recent studies have demonstrated elevated glucose levels before or at the time of diagnosis, which may play a role in the pathogenesis and progression of cancer.[7] Carcinogenesis in HNSCC is driven by diverse signalling pathways, however, little is known about the role of metabolism,[8] despite metabolic reprogramming being a recognised hallmark of cancer.[9]

T2D is a common condition characterised by insulin resistance and hyperglycaemia, which results in whole body metabolic dysregulation.[10] In 2015, the estimated worldwide diabetes prevalence for adults was predicted to rise to 642 million by 2040[11]. In addition, public health strategies have not successfully addressed the current 'obesity pandemic'.[12-16] One large pooled analysis demonstrated a weak positive association between T2D and HNSCC (OR 1.09; 95% confidence interval (95%CI): 0.95-1.24), which was stronger amongst those who never smoked cigarettes (OR 1.59; 95%CI: 1.22-2.07).[6] While obesity is a potent risk factor for T2D, the relationship between body mass index (BMI) and HNSCC is not straightforward, with both positive and inverse associations observed.[17] Another study found that a positive association

with BMI was only observed in never smokers.[18] In the largest study to date, waist circumference (WC) and waist-to-hip ratio (WHR) were positively associated with higher HNSCC risk, regardless of smoking status.[18] Metabolic syndrome has been described as a clustering of disorders including obesity, hypertension, hyperglycaemia and dyslipidemia. A moderate inverse association has been observed between metabolic syndrome and HNSCC (OR 0.81; 95%CI, 0.78-0.85), but again these results were modified by tobacco use.[19] Treatment for these conditions using medications such as statins,[20] or metformin,[21] may also reduce the risk of developing HNSCC. Given the growing pandemic of metabolic disorders, understanding how these alterations affect the risk of carcinogenesis may identify those who are high risk. This could drive targeted prevention, earlier detection and perhaps the identification or repurposing of drug targets for treatment in HNSCC.[22]

Rationale

The complex metabolic changes associated with T2D, obesity, dyslipidemia and hypertension may alter the risk of certain cancers, but the evidence for head and neck cancer is inconclusive. This study aims to identify, collate and synthesise all relevant studies including adult participants, to determine whether the risk of developing incident HNSCC is influenced by T2D, obesity, dyslipidemia and hypertension, using relevant effect measures (e.g., odds or risk ratios). Where available, we will further stratify by subsite (i.e., oral cavity, oropharynx and larynx), as well as by HPV status.

Methods

Research Question

‘Do metabolic disorders affect the risk of developing head and neck cancer?’

Study Design

Randomised controlled trials are informative for assessing causal effects, however due to the nature of the exposures in this study, inclusion of this design are not feasible. Therefore, we will focus on observational (e.g., cohort studies) reporting the risk of metabolic disorders on incident head and neck squamous cell carcinoma.

Eligibility criteria

Population- participants over 18 years old, of either sex, from any ethnic background.

Exposures- 1) type 2 diabetes, 2) obesity, 3) dyslipidemia and 4) hypertension. These will be collectively described as 'metabolic disorders.

Comparison- participants who have not been diagnosed with the aforementioned metabolic disorders.

Outcome- incident head and neck squamous cell carcinoma.

Table 1. Study selection criteria

Inclusion criteria	Exclusion criteria
• All studies published from January 1966 in the English language.<br>• Participants humans >18 years old, of either sex and any ethnic background.<br>• Observational (e.g., cohort studies) reporting the risk of incident head and neck squamous cell carcinoma.<br>• Studies must report an odds ratio (OR) or risk ratio (RR), or data which will allow these to be calculated.<br>• Exposures: Type 2 diabetes, obesity, dyslipidemia and hypertension with definitions as described in the study protocol.<br>• An outcome of head and neck cancer diagnosis which may be human papilloma virus (HPV) positive or negative. High risk types HPV16, 18, 31, and 33 only will be included.	• The study is not based on incident head and neck squamous cell carcinoma, or contains only prevalent data, meaning a temporal relationship cannot be inferred.<br>• Studies focused on cancer survival or progression.<br>• Study designs such as case reports or case series. In addition, cross-sectional, case-control and narrative review studies will be excluded due to the inability to infer a temporal relationship.<br>• Human studies only, no *in vivo* animal or *in vitro* cell line studies.<br>• Studies of head and neck epithelial dysplasia, potentially malignant disease or carcinoma *in situ*.<br>• Squamous cell carcinoma of other sites e.g., nasopharynx, salivary gland, oesophagus, skin or lung due to differing aetiology and risk factors e.g., Epstein Barr Virus.

For peer review only - http://bmjopen.bmj.com/site/about/guidelines.xhtml

The study will report in line with the Preferred Reporting Items for Systematic Reviews and Meta-Analyses (PRISMA) statement and has been pre-registered on International Prospective Register of Systematic Reviews (PROSPERO) in 2021 (CRD42021250520).

Search strategy

A systematic search strategy (**Supplementary Table 1**) has been formulated by clinicians, scientific researchers and a specialist librarian for Surgery, Head & Neck and Medicine. Medical Subject Headings (MeSH) and keywords will be iteratively combined with the Boolean operators AND, OR and NOT. Multiple databases will be searched from January 1966, including Cochrane Library, OVID SP versions of Medline and EMBASE. Pre-print servers including medRxiv and bioRxiv will also be searched. In addition, the following electronic bibliographic databases will be searched: EThOS, Google Scholar, Open Grey and ClinicalTrials.gov, to identify articles from the grey literature and conference proceedings. References extracted from the full-length articles will be reviewed to identify other publications of interest. Duplicate articles will be removed using Covidence©[23].

Data management

The results from searches will be imported into Covidence© software, to improve reference management and workflow. This will also populate a PRISMA flow diagram.[24]

Data screening and extraction

All titles and abstracts will be screened by two authors A.G. and C.R., with conflicts resolved by A.G., C.R. and M.G. Screening records and decisions will be kept in Covidence©. Data will be extracted from titles that meet the inclusion criteria **(Table 1)**. The data extraction form **(Supplementary Table 2)** will be piloted using 5 included titles with A.G., C.R. and M.G. independently completing data extraction. If amendments are necessary they will be

For peer review only - http://bmjopen.bmj.com/site/about/guidelines.xhtml

performed at this point, with consensus approval required prior to final use of the form. Subsequent to this, C.R. and M.G. will double data extract indepdently for each title, any disagreements will be discussed between A.G., C.R. and M.G. with consensus approval required.

Disease definitions:

1) The World Health Organization (WHO) defines T2D as a chronic disease that occurs when the body cannot effectively use the insulin it produces and is largely the result of excess body weight and physical inactivity. A diagnosis of T2D is defined as symptoms such as polyuria or polydipsia, plus:[25, 26]

- a random blood plasma glucose concentration ≥ 11.1 mmol/l or;
- a fasting plasma glucose concentration ≥ 7.0 mmol/l (whole blood ≥ 6.1 mmol/l) or;
- two hour plasma glucose concentration ≥ 11.1 mmol/l two hours after 75g anhydrous glucose in an oral glucose tolerance test (OGTT) or;
- glycated haemoglobin (HbA1c) 6.5% or more (48 mmol/mol and above)

2) The World Health Organization (WHO) defines obesity as having: a body mass index (BMI) of 30 or above.[27]

3) Dyslipidemia is classified as serum total cholesterol (TC), low density lipoprotein cholesterol (LDL-C), triglycerides, apolipoprotein B, or lipoprotein(a) concentrations above the 90th percentile, or high density lipoprotein cholesterol (HDL-C) or apolipoprotein concentrations below the 10th percentile for the general population.[28]

4) The World Health Organization (WHO) defines hypertension as:[29]

For peer review only - http://bmjopen.bmj.com/site/about/guidelines.xhtml

- a systolic blood pressure reading of ≥140 mmHg when recorded on two different days; and/or
- a diastolic blood pressure readings on both days of ≥90 mmHg.

A diagnosis of incident head and neck squamous cell carcinoma should be confirmed by histology by a trained pathologist. Cancer diagnosis and confirmation may be reported using the International Classification of Diseases (ICD) codes.[30] Cancer cases for the disease of interest have the following ICD codes: oral cavity (C02.0-C02.9, C03.0-C03.9, C04.0-C04.9, C05.0-C06.9) oropharynx (C01.9, C02.4, C09.0-C10.9), hypopharynx (C13.0-C13.9), overlapping (C14 and combination of other sites) and 25 cases with unknown ICD code (other). With respect to HPV diagnosis, only high risk subtypes HPV16, 18, 31, and 33 will be included. These are should be detected using HPV-DNA via polymerase chain reaction (PCR) and/or *in situ* hybridization (ISH) examination, usually used in combination with immunohistochemistry for *p16*.

Data analysis and synthesis

The analysis and synthesis process will be performed in two phases. The first phase consists of estimating the effect of each metabolic disorder (T2D, obesity, dyslipidemia and hypertension) on incident HNSCC separately for each included study. Effects should be reported as odds (OR) or risk ratios (RR), with 95% confidence intervals or in a format that will enable us to calculate these.

The second phase will focus on estimating the combined effect across studies using meta-analysis, using metafor software in R (R Core Team, version 4.0.3).[31] The analysis will yield information about the heterogeneity of effects across studies, using Cochran's Q test and Higgins' I^2 statistic. Due to the heterogeneity in study design, exposure definition and outcome reporting in the included studies, meta-analysis may not be reasonable.

For peer review only - http://bmjopen.bmj.com/site/about/guidelines.xhtml

Subgroup analyses

Where possible, we will stratify by oral, oropharyngeal and laryngeal subsite and by HPV-status as post-hoc analyses, to determine if effects seen are specific to a particular anatomical region or associated with specific tumour types.

Risk of bias in studies

Two authors will independently extract the relevant risk factor information during data extraction using a preliminary version of the Risk of Bias in Non-randomised Studies – of Exposures (ROBINS-E) tool (https://www.bristol.ac.uk/population-health-sciences/centres/cresyda/barr/riskofbias/robins-e/).[32] The ROBINS-E tool aims to assess the result of interest from an observational study for risk of bias, where that study is designed to assess the effect of exposure on outcome.[32] Disagreements will be recorded and discussed with a third author for resolution. This assessment will be used in evaluating the strength of evidence from the included studies.

Amendments

Any amendments to the initial protocol will be included in an update to the PROSPERO record, including information on amendment type, reasoning and a timestamp. Deviation from this protocol will be described in the full systematic review paper.

Dissemination

This systematic review will be published in a relevant peer-reviewed journal and findings from this work may be presented at scientific meetings (both poster and oral presentations).

Ethical approval

No ethical approval is required for the conducting of this systematic review.

Acknowledgements

None to declare.

For peer review only - http://bmjopen.bmj.com/site/about/guidelines.xhtml

References

1. Cancer Research UK (CRUK). Head and neck cancer statistics 2019 [cited 2019 11/04/2019]. Available from: https://www.cancerresearchuk.org/health-professional/cancer-statistics/statistics-by-cancer-type/head-and-neck-cancers#heading-Two accessed 13/09/2021 2019.

2. Thomas SJ, Penfold CM, Waylen A, et al. The changing aetiology of head and neck squamous cell cancer: A tale of three cancers? *Clin Otolaryngol* 2018;43(4):999-1003. doi: 10.1111/coa.13144

3. Office for National Statistics. Data for 1974 onwards: Office for National Statistics. Adult Smoking habits in Great Britain. 2020 [Available from: https://www.ons.gov.uk/peoplepopulationandcommunity/healthandsocialcare/drugusealcoholandsmoking/datasets/adultsmokinghabitsingreatbritain. accessed 01/03/2021 2020.

4. Office for National Statistics. Adult drinking habits in Great Britain 2017 [Available from: https://www.ons.gov.uk/peoplepopulationandcommunity/healthandsocialcare/drugusealcoholandsmoking/datasets/adultdrinkinghabits accessed 01/03/2021 2021.

5. Wotton CJ, Yeates DG, Goldacre MJ. Cancer in patients admitted to hospital with diabetes mellitus aged 30 years and over: record linkage studies. *Diabetologia* 2011;54(3):527-34. doi: 10.1007/s00125-010-1987-2 [published Online First: 2010/12/01]

6. Stott-Miller M, Chen C, Chuang SC, et al. History of diabetes and risk of head and neck cancer: a pooled analysis from the international head and neck cancer epidemiology consortium. *Cancer Epidemiol Biomarkers Prev* 2012;21(2):294-304. doi: 10.1158/1055-9965.EPI-11-0590 [published Online First: 2011/12/07]

7. Vilaseca I, Fuster G, Avilés-Jurado FX. The impact of diabetes in head and neck cancer. *Curr Opin Otolaryngol Head Neck Surg* 2020;28(2):107-11. doi: 10.1097/moo.0000000000000606 [published Online First: 2020/02/06]

8. Rego DF, Elias ST, Amato AA, et al. Anti-tumor effects of metformin on head and neck carcinoma cell lines: A systematic review. *Oncol Lett* 2017;13(2):554-66. doi: 10.3892/ol.2016.5526 [published Online First: 2017/03/31]

9. Hanahan D, Weinberg RA. Hallmarks of Cancer: The Next Generation. *Cell* 2011;144(5):646-74. doi: 10.1016/j.cell.2011.02.013

10. Koivula RW, Heggie A, Barnett A, et al. Discovery of biomarkers for glycaemic deterioration before and after the onset of type 2 diabetes: rationale and design of the epidemiological studies within the IMI DIRECT Consortium. *Diabetologia* 2014;57(6):1132-42. doi: 10.1007/s00125-014-3216-x [published Online First: 2014/04/04]

11. Ogurtsova K, da Rocha Fernandes JD, Huang Y, et al. IDF Diabetes Atlas: Global estimates for the prevalence of diabetes for 2015 and 2040. *Diabetes Res Clin Pract* 2017;128:40-50. doi: 10.1016/j.diabres.2017.03.024 [published Online First: 2017/04/25]

12. Wolf AM, Colditz GA. Current estimates of the economic cost of obesity in the United States. *Obes Res* 1998;6(2):97-106. doi: DOI 10.1002/j.1550-8528.1998.tb00322.x

13. Dombrowski SU, Knittle K, Avenell A, et al. Long term maintenance of weight loss with non-surgical interventions in obese adults: systematic review and meta-analyses of randomised controlled trials. *Bmj-Brit Med J* 2014;348 doi: ARTN g2646
10.1136/bmj.g2646

For peer review only - http://bmjopen.bmj.com/site/about/guidelines.xhtml

14. Gray LJ, Cooper N, Dunkley A, et al. A systematic review and mixed treatment comparison of pharmacological interventions for the treatment of obesity. *Obes Rev* 2012;13(6):483-98. doi: 10.1111/j.1467-789X.2011.00981.x

15. Yanovski SZ, Yanovski JA. Long-term Drug Treatment for Obesity A Systematic and Clinical Review. *Jama-J Am Med Assoc* 2014;311(1):74-86. doi: 10.1001/jama.2013.281361

16. Corbin LJ, Timpson NJ. Body Mass Index: Has Epidemiology Started to Break Down Causal Contributions to Health and Disease? *Obesity* 2016;24(8):1630-38. doi: 10.1002/oby.21554

17. Maasland DH, van den Brandt PA, Kremer B, et al. Body mass index and risk of subtypes of head-neck cancer: the Netherlands Cohort Study. *Sci Rep* 2015;5:17744. doi: 10.1038/srep17744 [published Online First: 2015/12/05]

18. Gaudet MM, Kitahara CM, Newton CC, et al. Anthropometry and head and neck cancer:a pooled analysis of cohort data. *Int J Epidemiol* 2015;44(2):673-81. doi: 10.1093/ije/dyv059 [published Online First: 2015/06/08]

19. Stott-Miller M, Chen C, Schwartz SM. Type II diabetes and metabolic syndrome in relation to head and neck squamous cell carcinoma risk: a SEER-Medicare database study. *Cancer Epidemiol* 2013;37(4):428-33. doi: 10.1016/j.canep.2013.03.006 [published Online First: 2013/04/09]

20. Kao LT, Hung SH, Kao PF, et al. Inverse association between statin use and head and neck cancer: Population-based case-control study in Han population. *Head Neck-J Sci Spec* 2019;41(5):1193-98. doi: 10.1002/hed.25501

21. Yen YC, Lin C, Lin SW, et al. Effect of metformin on the incidence of head and neck cancer in diabetics. *Head Neck* 2015;37(9):1268-73. doi: 10.1002/hed.23743 [published Online First: 2014/05/08]

22. Ramteke P, Deb A, Shepal V, et al. Hyperglycemia Associated Metabolic and Molecular Alterations in Cancer Risk, Progression, Treatment, and Mortality. *Cancers (Basel)* 2019;11(9) doi: 10.3390/cancers11091402 [published Online First: 2019/09/25]

23. Covidence. Covidence - Better systematic review management 2021 [Available from: https://www.covidence.org/ accessed 13/09/2021 2021.

24. Liberati A, Altman DG, Tetzlaff J, et al. The PRISMA statement for reporting systematic reviews and meta-analyses of studies that evaluate healthcare interventions: explanation and elaboration. *BMJ* 2009;339:b2700. doi: 10.1136/bmj.b2700

25. World Health Organization. Definition and Diagnosis of Diabetes Mellitus and Intermediate Hyperglycaemia 2006 [Available from: https://www.diabetes.org.uk/resources-s3/2017-09/Definition_and_diagnosis1111.pdf accessed 01/03/2021 2021.

26. World Health Organization. Use of Glycated Haemoglobin (HbA1c) in the Diagnosis of Diabetes Mellitus 2011 [Available from: https://www.diabetes.org.uk/resources-s3/2017-09/Definition_and_diagnosis1111.pdf accessed 01/03/2021 2021.

27. World Health Organization. Obesity and overweight 2021 [Available from: https://www.who.int/news-room/fact-sheets/detail/obesity-and-overweight accessed 01/03/2021 2021.

28. British Medical Journal. Hypercholesterolemia 2019 [Available from: https://bestpractice.bmj.com/topics/en-us/170 accessed 28.03.2021 2021.

29. Organization WH. Hypertension 2021 [Available from: https://www.who.int/news-room/fact-sheets/detail/hypertension accessed 13/09/2021 2021.

30. Organization WH. International Statistical Classification of Diseases and Related Health Problems (ICD) 2021 [accessed 13/09/2021 2021.

31. Viechtbauer W. metafor: Meta-Analysis Package for R 2021 [Available from: https://cran.r-project.org/web/packages/metafor/index.html accessed October 2021 October 2021.

32. Bristol Uo. The ROBINS-E tool (Risk Of Bias In Non-randomized Studies - of Exposures) 2021 [Available from: http://www.bristol.ac.uk/population-health-sciences/centres/cresyda/barr/riskofbias/robins-e/ accessed 13/09/2021 2021.

Authors' contributions

AG and CR jointly contributed to the development of the protocol and the drafting, writing and editing of this manuscript. FS contributed to the development of this study and editing of the manuscript. JH contributed to the development of the search strategy. BM, EG, EV, RR and JPTH contributed to the development of this study. MG was responsible for conceptualising this study, drafting and editing this manuscript. All authors contributed to the development of the search strategy. All authors have approved and contributed to the final written manuscript.

Funding statement

A.G. is a National Institute for Health Research (NIHR) academic clinical fellow (no grant number). F.S. was supported by a Cancer Research UK (C18281/A29019) programme grant (the Integrative Cancer Epidemiology Programme). E.E.V is supported by Diabetes UK (17/0005587). E.E.V is also supported by the World Cancer Research Fund (WCRF UK), as part of the World Cancer Research Fund International grant programme (IIG_2019_2009). R.C.R. is a de Pass VC research fellow at the University of Bristol (no grant number). J.P.T.H supported by the NIHR Biomedical Research Centre at University Hospitals Bristol and Weston NHS Foundation Trust and the University of Bristol (no grant number), the NIHR Applied Research Collaboration West at University Hospitals Bristol and Weston NHS Foundation Trust (no grant number) and is a National Institute for Health Research (NIHR) Senior Investigator (no grant number). M.G. is currently supported by a Wellcome Trust GW4-Clinical Academic Training PhD Fellowship. This research was funded in part, by the Wellcome Trust [Grant number 220530/Z/20/Z]. For the purpose of Open Access, the author has applied a CC BY public copyright licence to any Author Accepted Manuscript version arising from this submission. The views expressed in this publication are those of the author(s) and not necessarily those of Wellcome, NIHR, the NHS or Department of Health.

Competing interests statement

None declared

Patient and Public Involvement

Patients and/or the public were not involved in the development, conduct or proposed dissemination of this systematic review protocol.

Word Count: 1,755 (excluding abstract)

Supplementary Table 1. Search strategy

Supplementary Table 2. Data extraction table. Note separate table for each exposure (Obesity, Dyslipidaemia, Hypertension, Type 2 diabetes).

Supplementary Checklist 1. PRISMA-P checklist.

Supplementary Table 1. Search strategy

1
exp "HEAD AND NECK NEOPLASMS"/
2
("head and neck cancer" or "oral cancer").ti,ab.
3
((lip or lips or mouth or gums or palatal or palate or gingiva* or buccal or cheek* or tongue or lingual or glosso* or laryn* or "vocal chord*" or "vocal cord*" or "vocal fold*" or subglottic or supraglottic or hypopharyn* or hypo-pharyn* or epiglottis or arytenoids or aryepiglottic or throat or oropharyn* or oro-pharyn* or tonsil* or pharyn* or otolaryn* or jaw or mandib* or maxill*) adj3 (cancer* or carcinoma* or neoplasm* or tumour* or tumor* or squamous or lymphoma* or HPV or "human papillomavirus")).ti,ab.
4
1 or 2 or 3
5
exp DYSLIPIDEMIAS/
6
(dyslipidaemia or dyslipidemia or lipolysis or hypercholesterolemia or hypercholesterolaemia or "low density lipoprotein" or “apolipoprotein” or cholesterol or LDL or triglycerides or statin* or ezetimibe or "HMG-CoA" or "reductase inhibitor*" or "HMGCR" or "hydroxymethylglutaryl coa reductases" or "NPC1L1" or "PCSK9" or "CETP" or "LDLR" or atorvastatin or fluvastatin or lovastatin or pitavastatin or pravastatin or rosuvastatin or simvastatin or (lipid* adj2 (metaboli* or medicat* or drug* or lower* or reduc* or decreas*))).ti,ab.
7
5 or 6
8
exp DIABETES MELLITUS/
9
(Diabet* or insulin or "insulin resistance" or hyperglycaemia or hypoglycaemia or hyperglycemia or hypoglycemia or hypertension or metformin or glucagon or "hypoglycaemic agent*" or HbA1 or HbA1c or (glucose adj2 (impair* or tolerance or intolerance)) or IGT or glycogenolysis or gluconeogenesis or GDF?15 or gliflozins or SGLT2 or sulphonylurea* or meglitinide* or thiazolidinediones or "DPP-4 inhibitor*" or "GLP-1 receptor agonist*").ti,ab.
10
8 or 9
11
exp METABOLIC SYNDROME/
12
(metabolic syndrome or "dyresgulated metaboli*" or obesity or obes* or "abdominal obes*" or (fat or fatty or fats) or "body weight" or adipos* or (weight adj2 gain*) or overeat* or bariatric or (over adj2 eat) or "body mass index*" or BMI).ti,ab.
13
11 or 12
14
7 or 10 or 13
15
4 and 14
16
exp RISK/
17
(risk* or inciden* or prevalen*).ti,ab.
18
16 or 17
19
15 and 18

For peer review only

Supplementary Table 2. Data extraction table. Note separate table for each exposure (Obesity, Dyslipidaemia, Hypertension, Type 2 diabetes).

Obesity

Study ID	First Author	Year	Study design	Sample size	Age in years (mean)	Age in years (SD)	Age in years (median)	Age in years (IQR)	Age in years (range)
Other measures of age	Sex (% of female)	Sex (% of male)	White	Black	Hispanic	Other	Ethnicity	Country	Population setting
Method of exposure diagnosis	Exposure (units)	Treatment taken for exposure	Method of outcome assessment	HPV status	Adjusted covariates	Outcome (subsite)	no. with obesity and outcome	Reported Statistic	Effect size
95% CI	Follow-up in years (mean)	Follow-up in years (SD)	Follow-up in years (median)	Follow-up in years (IQR)	Follow-up in years (range)	Other measures of follow-up in years			

Dyslipidaemia

Study ID	First Author	Year	Study design	Sample size	Age in years (mean)	Age in years (SD)	Age in years (median)	Age in years (IQR)	Age in years (range)
Other measures of age	Sex (% of female)	Sex (% of male)	White	Black	Hispanic	Other	Ethnicity	Country	Population setting
Exposure (units)	Method of exposure diagnosis	Treatment taken for exposure	Method of outcome assessment	HPV status	Adjusted covariates	Outcome (subsite)	no. with dyslipidaemia and outcome	Reported Statistic	Effect size
95% CI	Follow-up in years (mean)	Follow-up in years (SD)	Follow-up in years (median)	Follow-up in years (IQR)	Follow-up in years (range)	Other measures of follow-up in years			

Hypertension

Study ID	First Author	Year	Study design	Sample size	Age in years (mean)	Age in years (SD)	Age in years (median)	Age in years (IQR)	Age in years (range)
Other measures of age	Sex (% of female)	Sex (% of male)	White	Black	Hispanic	Other	Ethnicity	Country	Population setting
Exposure (units)	Method of exposure diagnosis	Treatment taken for exposure	Method of outcome assessment	HPV status	Adjusted covariates	Outcome (subsite)	no. with hypertension and outcome	Reported Statistic	Effect size
95% CI	Follow-up in years (mean)	Follow-up in years (SD)	Follow-up in years (median)	Follow-up in years (IQR)	Follow-up in years (range)	Other measures of follow-up in years			

For peer review only

Type 2 diabetes

Study ID	First Author	Year	Study design	Sample size	Age in years (mean)	Age in years (SD)	Age in years (median)	Age in years (IQR)	Age in years (range)
Other measures of age	Sex (% of female)	Sex (% of male)	White	Black	Hispanic	Other	Ethnicity	Country	Population setting
Exposure (units)	Method of exposure diagnosis	Treatment taken for exposure	Method of outcome assessment	HPV status	Adjusted covariates	Outcome (subsite)	no. with type 2 diabetes and outcome	Reported Statistic	Effect size
95% CI	Follow-up in years (mean)	Follow-up in years (SD)	Follow-up in years (median)	Follow-up in years (IQR)	Follow-up in years (range)	Other measures of follow-up in years			

For peer review only

Supplementary Checklist 1. PRISMA-P checklist.

Reporting checklist for protocol of a systematic review and meta analysis.

Instructions to authors

Complete this checklist by entering the page numbers from your manuscript where readers will find each of the items listed below.

Your article may not currently address all the items on the checklist. Please modify your text to include the missing information. If you are certain that an item does not apply, please write "n/a" and provide a short explanation.

Upload your completed checklist as an extra file when you submit to a journal.

In your methods section, say that you used the PRISMA-Preporting guidelines, and cite them as:

Moher D, Shamseer L, Clarke M, Ghersi D, Liberati A, Petticrew M, Shekelle P, Stewart LA. Preferred Reporting Items for Systematic Review and Meta-Analysis Protocols (PRISMA-P) 2015 statement. Syst Rev. 2015;4(1):1.

		Reporting Item	Page Number
**Title**			
Identification	#1a	Identify the report as a protocol of a systematic review	1
Update	#1b	If the protocol is for an update of a previous systematic review, identify as such	N/A
**Registration**			
	#2	If registered, provide the name of the registry (such as PROSPERO) and registration number	8
**Authors**			
Contact	#3a	Provide name, institutional affiliation, e-mail address of all protocol authors; provide physical mailing address of corresponding author	1
Contribution	#3b	Describe contributions of protocol authors and identify the guarantor of the review	16
**Amendments**			
	#4	If the protocol represents an amendment of a previously completed or published protocol, identify as such and list changes; otherwise, state plan for documenting important protocol amendments	N/A

For peer review only

**Support**			
Sources	#5a	Indicate sources of financial or other support for the review	16
Sponsor	#5b	Provide name for the review funder and / or sponsor	16
Role of sponsor or funder	#5c	Describe roles of funder(s), sponsor(s), and / or institution(s), if any, in developing the protocol	16
**Introduction**			
Rationale	#6	Describe the rationale for the review in the context of what is already known	6
Objectives	#7	Provide an explicit statement of the question(s) the review will address with reference to participants, interventions, comparators, and outcomes (PICO)	7
**Methods**			
Eligibility criteria	#8	Specify the study characteristics (such as PICO, study design, setting, time frame) and report characteristics (such as years considered, language, publication status) to be used as criteria for eligibility for the review	7
Information sources	#9	Describe all intended information sources (such as electronic databases, contact with study authors, trial registers or other grey literature sources) with planned dates of coverage	8
Search strategy	#10	Present draft of search strategy to be used for at least one electronic database, including planned limits, such that it could be repeated	18
Study records - data management	#11a	Describe the mechanism(s) that will be used to manage records and data throughout the review	8
Study records - selection process	#11b	State the process that will be used for selecting studies (such as two independent reviewers) through each phase of the review (that is, screening, eligibility and inclusion in meta-analysis)	8-9
Study records - data collection process	#11c	Describe planned method of extracting data from reports (such as piloting forms, done independently, in duplicate), any processes for obtaining and confirming data from investigators	8-9
Data items	#12	List and define all variables for which data will be sought (such as PICO items, funding sources), any pre-planned data assumptions and simplifications	19
Outcomes and prioritization	#13	List and define all outcomes for which data will be sought, including prioritization of main and additional outcomes, with rationale	10
Risk of bias in individual studies	#14	Describe anticipated methods for assessing risk of bias of individual studies, including whether this will be done at the	11

For peer review only

		outcome or study level, or both; state how this information will be used in data synthesis	
Data synthesis	#15a	Describe criteria under which study data will be quantitatively synthesised	10-11
Data synthesis	#15b	If data are appropriate for quantitative synthesis, describe planned summary measures, methods of handling data and methods of combining data from studies, including any planned exploration of consistency (such as I2, Kendall's τ)	10-11
Data synthesis	#15c	Describe any proposed additional analyses (such as sensitivity or subgroup analyses, meta-regression)	10-11
Data synthesis	#15d	If quantitative synthesis is not appropriate, describe the type of summary planned	N/A
Meta-bias(es)	#16	Specify any planned assessment of meta-bias(es) (such as publication bias across studies, selective reporting within studies)	11
Confidence in cumulative evidence	#17	Describe how the strength of the body of evidence will be assessed (such as GRADE)	11

The PRISMA-P elaboration and explanation paper is distributed under the terms of the Creative Commons Attribution License CC-BY. This checklist was completed on 13. September 2021 using https://www.goodreports.org/, a tool made by the EQUATOR Network in collaboration with Penelope.ai

For peer review only

BMJ Open

Metabolic disorders and the risk of head and neck cancer: a protocol for a systematic review and meta-analysis

Journal:	*BMJ Open*
Manuscript ID	bmjopen-2021-058392.R1
Article Type:	Protocol
Date Submitted by the Author:	22-Mar-2022

Complete List of Authors:	Gormley, Alexander; University of Bristol, Dental School, Faculty of Health Sciences; Bristol Dental Hospital, University Hospitals Bristol and Weston NHS Foundation Trust<br>Richards, Charlotte; Cardiff University, School of Dentistry<br>Spiga, Francesca; University of Bristol, Population Health Sciences, Bristol Medical School, Faculty of Health Sciences; University of Bristol, MRC Integrative Epidemiology Unit, Population Health Sciences, Bristol Medical School, Faculty of Health Sciences<br>Gray, Emily; University Hospitals Bristol and Weston NHS Foundation Trust<br>Hooper, Joanna; Library and information services, University Hospitals Bristol and Weston NHS Foundation Trust<br>Main, Barry; University of Bristol, Bristol Dental School, Faculty of Health Sciences, University of Bristol; University of Bristol, Population Health Sciences, Faculty of Health Sciences<br>Vincent, Emma; University of Bristol, MRC Integrative Epidemiology Unit, Population Health Sciences, Bristol Medical School, Faculty of Health Sciences; University of Bristol, School of Cellular and Molecular Medicine, Faculty of Life Sciences<br>Richmond, Rebecca; University of Bristol, Population Health Sciences, Bristol Medical School, Faculty of Health Sciences; University of Bristol, MRC Integrative Epidemiology Unit, Population Health Sciences, Bristol Medical School, Faculty of Health Sciences<br>Higgins, Julian; University of Bristol, Population Health Sciences, Bristol Medical School, Faculty of Health Sciences; University of Bristol, MRC Integrative Epidemiology Unit, Population Health Sciences, Bristol Medical School, Faculty of Health Sciences<br>Gormley, Mark; University of Bristol, Bristol Dental School, Faculty of Health Sciences; University of Bristol, MRC Integrative Epidemiology Unit, Population Health Sciences, Bristol Medical School, Faculty of Health Sciences

**Primary Subject Heading**:	Oncology
Secondary Subject Heading:	Nutrition and metabolism, Oncology, Dentistry and oral medicine, Research methods
Keywords:	ONCOLOGY, ORAL & MAXILLOFACIAL SURGERY, Head & neck tumours < ONCOLOGY, Hypertension < CARDIOLOGY, Lipid disorders < DIABETES & ENDOCRINOLOGY, DIABETES & ENDOCRINOLOGY

SCHOLARONE™
Manuscripts

Metabolic disorders and the risk of head and neck cancer: a protocol for a systematic review and meta-analysis

Alexander Gormley[1,2], Charlotte Richards[3*], Francesca Spiga[4,5], Emily Gray[2], Joanna Hooper[6], Barry G Main[1,2,4], Emma E Vincent[4,5,7], Rebecca C Richmond[4,5], Julian PT Higgins[4,5], Mark Gormley[1,2,4,5]*

1 – Bristol Dental School, Faculty of Health Sciences, University of Bristol

2 – Bristol Dental Hospital, University Hospitals Bristol and Weston NHS Foundation Trust

3 – School of Dentistry, Cardiff University

4 – Population Health Sciences, Bristol Medical School, Faculty of Health Sciences, University of Bristol

5 – MRC Integrative Epidemiology Unit, Population Health Sciences, Bristol Medical School, Faculty of Health Sciences, University of Bristol

6 – Library and information services, University Hospitals Bristol and Weston NHS Foundation Trust

7 – School of Cellular and Molecular Medicine, Faculty of Life Sciences, University of Bristol

* - Joint first authors

Corresponding author – mark.gormley@bristol.ac.uk

Mark Gormley, Bristol Dental School, University of Bristol, Lower Maudlin Street, Bristol, BS1 2LY.

Abstract

Introduction

Head and neck cancer squamous cell carcinoma (HNSCC) is the 6th most common cancer internationally. Established risk factors include smoking, alcohol and presence of human papillomavirus (HPV). The incidence rate of new disease continues to rise, despite falls in alcohol consumption and a reduction in smoking, the rising rates are unlikely to be solely attributed to HPV status alone. Obesity and its associated conditions such as type 2 diabetes (T2D) are implicated in the risk and progression of a variety of cancers but there is paucity of evidence regarding its role in HNSCC.

Methods and analysis

A systematic review of cohort studies, reporting a risk of incident head and neck squamous cell carcinoma will be included. A systematic search strategy has been developed, multiple databases will be searched from January 1966 – November 2021, including Cochrane Library, OVID SP versions of Medline and EMBASE. The primary outcome will be incident HNSCC based on exposures of type 2 diabetes, obesity, dyslipidemia and hypertension as defined by the World Health Organisation (WHO). A combined risk effect across studies will be calculated using meta-analysis, although depending on the heterogeneity in study design, exposure and outcome reporting this may not be possible.

Ethics and dissemination

No ethical approval is required for this systematic review. The review will be published in a revelant peer-review journal and findings will be presented at scientific meetings in both poster and oral presentation form.

For peer review only - http://bmjopen.bmj.com/site/about/guidelines.xhtml

Registration details

This study has been registered with the international prospective register of systematic reviews (PROSPERO) with study registration number CRD42021250520. This protocol has been developed in accordance with the preferred reporting items for systematic review and meta-analysis protocols (PRISMA-P) guidance statement.

For peer review only - http://bmjopen.bmj.com/site/about/guidelines.xhtml

Article Summary

Strengths and limitations of this study

- This systematic review will be the first to comprehensively review the literature and provide a meta-analysis of the effect of metabolic disorders and the risk of incident HNSCC.
- The publication of this protocol provides a clear representation of the methods used in this review for transparency and to prevent future duplication.
- This systematic review will be one of the first to use the The ROBINS-E tool (Risk Of Bias In Non-randomised Studies - of Exposures).
- Despite the metabolic disorders being described as separate entities, the authors recognise the potential for disease processes to be related.
- Among cohort studies there is significant variability in terms of length of follow-up for the outcome of interest, HNSCC.

For peer review only - http://bmjopen.bmj.com/site/about/guidelines.xhtml

Introduction

Head and neck squamous cell carcinoma (HNSCC), which includes cancer of the oral cavity, oropharynx, hypopharynx and larynx, is the world's 6th most common cancer, with the highest incidence in males and those over 70 years old.[1] Established risk factors include smoking, alcohol and the human papillomavirus (HPV), which has mainly been linked to oropharyngeal cancer.[2] Despite the significant reduction in smoking,[3] and a fall in alcohol consumption,[4] in the UK over the previous two decades, incidence rates of head and neck cancers have continued to rise by around a third.[1] Given this potentially changing aetiology,[2] further exploration of less well known risk factors is warranted to target prevention and identify patients with early disease. People living with obesity and associated conditions such as type 2 diabetes (T2D), have an increased risk of developing certain cancers (e.g. liver and pancreas), but the epidemiological evidence surrounding HNSCC is not conclusive.[5 6] Recent studies have demonstrated elevated glucose levels before or at the time of diagnosis, which may play a role in the pathogenesis and progression of cancer.[7] Carcinogenesis in HNSCC is driven by diverse signalling pathways, however, little is known about the role of metabolism,[8] despite metabolic reprogramming being a recognised hallmark of cancer.[9]

T2D is a common condition characterised by insulin resistance and hyperglycaemia, which results in whole body metabolic dysregulation.[10] In 2015, the estimated worldwide diabetes prevalence for adults was predicted to rise to 642 million by 2040[11]. In addition, public health strategies have not successfully addressed the current 'obesity pandemic'.[12-16] One large pooled analysis demonstrated a weak positive association between T2D and HNSCC (OR 1.09; 95% confidence interval (95%CI): 0.95-1.24), which was stronger amongst those

For peer review only - http://bmjopen.bmj.com/site/about/guidelines.xhtml

who never smoked cigarettes (OR 1.59; 95%CI: 1.22-2.07).[6] While obesity is a potent risk factor for T2D, the relationship between body mass index (BMI) and HNSCC is not straightforward, with both positive and inverse associations observed.[17] Another study found that a positive association with BMI was only observed in never smokers.[18] In the largest study to date, waist circumference (WC) and waist-to-hip ratio (WHR) were positively associated with higher HNSCC risk, regardless of smoking status.[18] Metabolic syndrome has been described as a clustering of disorders including obesity, hypertension, hyperglycaemia and dyslipidemia. A moderate inverse association has been observed between metabolic syndrome and HNSCC (OR 0.81; 95%CI, 0.78-0.85), but again these results were modified by tobacco use.[19] Treatment for these conditions using medications such as statins,[20] or metformin,[21] may also reduce the risk of developing HNSCC. Given the growing pandemic of metabolic disorders, understanding how these alterations affect the risk of carcinogenesis may identify those who are high risk. This could drive targeted prevention, earlier detection and perhaps the identification or repurposing of drug targets for treatment in HNSCC.[22]

Rationale

The complex metabolic changes associated with T2D, obesity, dyslipidemia and hypertension may alter the risk of certain cancers, but the evidence for head and neck cancer is inconclusive. This study aims to identify, collate and synthesise all relevant studies including adult participants, to determine whether the risk of developing incident HNSCC is influenced by T2D, obesity, dyslipidemia and hypertension, using relevant effect measures (e.g., odds or risk ratios). Where available, we will further stratify by subsite (i.e., oral cavity, oropharynx, hypopharynx and larynx), as well as by HPV status.

For peer review only - http://bmjopen.bmj.com/site/about/guidelines.xhtml

Methods

Research Question

'Do metabolic disorders affect the risk of developing head and neck cancer?'

Study Design

Randomised controlled trials are informative for assessing causal effects, however due to the nature of the exposures in this study, inclusion of this design are not feasible. Therefore, we will focus on observational (e.g., cohort studies) reporting the risk of metabolic disorders on incident head and neck squamous cell carcinoma.

Eligibility criteria

Population- participants over 18 years old, of either sex, from any ethnic background.

Exposures- 1) type 2 diabetes, 2) obesity, 3) dyslipidemia and 4) hypertension. These will be collectively described as 'metabolic disorders.

Comparison- participants who have not been diagnosed with the aforementioned metabolic disorders.

Outcome- incident head and neck squamous cell carcinoma.

For peer review only - http://bmjopen.bmj.com/site/about/guidelines.xhtml

Table 1. Study selection criteria

Inclusion criteria	Exclusion criteria
• All studies published from January 1966 in the English language.<br>• Participants humans >18 years old, of either sex and any ethnic background.<br>• Observational (e.g., cohort studies) reporting the risk of incident head and neck squamous cell carcinoma.<br>• Studies must report an odds ratio (OR) or risk ratio (RR), or data which will allow these to be calculated.<br>• Exposures: Type 2 diabetes, obesity, dyslipidemia and hypertension with definitions as described in the study protocol.<br>• An outcome of head and neck cancer diagnosis which may be human papilloma virus (HPV) positive or negative. High risk types HPV16, 18, 31, and 33 only will be included.	• The study is not based on incident head and neck squamous cell carcinoma, or contains only prevalent data, meaning a temporal relationship cannot be inferred.<br>• Studies focused on cancer survival or progression.<br>• Study designs such as case reports or case series. In addition, cross-sectional, case-control and narrative review studies will be excluded due to the inability to infer a temporal relationship.<br>• Human studies only, no *in vivo* animal or *in vitro* cell line studies.<br>• Studies of head and neck epithelial dysplasia, potentially malignant disease or carcinoma *in situ*.<br>• Squamous cell carcinoma of other sites e.g., nasopharynx, salivary gland, oesophagus, skin or lung due to differing aetiology, histological subtypes and risk factors e.g., Epstein Barr Virus.

The study will report in line with the Preferred Reporting Items for Systematic Reviews and Meta-Analyses (PRISMA) statement and has been pre-registered on International Prospective Register of Systematic Reviews (PROSPERO) in 2021 (CRD42021250520).

Search strategy

A systematic search strategy (**Supplementary Table 1**) has been formulated by clinicians, scientific researchers and a specialist librarian for Surgery, Head & Neck and Medicine. Medical Subject Headings (MeSH) and keywords will be iteratively combined with the Boolean operators AND, OR and NOT. Multiple databases will be searched from January 1966 – November 2021, including OVID SP versions of Medline and EMBASE, using the

For peer review only - http://bmjopen.bmj.com/site/about/guidelines.xhtml

formulated search strategy (**Supplementary Table 1**). Pre-print servers including medRxiv and bioRxiv will also be searched. In addition, the following electronic bibliographic databases will be searched: Cochrane Library, EThOS, Google Scholar, Open Grey and ClinicalTrials.gov, to identify articles from the grey literature and conference proceedings using a modified search strategy (**Supplementary Table 2**). References extracted from the full-length articles will be reviewed to identify other publications of interest. Duplicate articles will be removed using Covidence©[23].

Data management

The results from searches will be imported into Covidence© software, to improve reference management and workflow. This will also populate a PRISMA flow diagram.[24]

Data screening and extraction

All titles and abstracts will be screened by two authors A.G. and C.R., with conflicts resolved by M.G. Screening records and decisions will be kept in Covidence©. Data will be extracted from titles that meet the inclusion criteria **(Table 1)**. The data extraction form **(Supplementary Table 3)** will be piloted using five included titles with A.G., C.R. and M.G. independently completing data extraction. If amendments are necessary they will be performed at this point, with consensus approval required prior to final use of the form. Subsequent to this, C.R. and M.G. will double data extract independently for each title, any disagreements will be discussed between A.G., C.R. and M.G. with consensus approval required.

Exposure definitions:

1) The World Health Organization (WHO) defines T2D as a chronic disease that occurs when the body cannot effectively use the insulin it produces and is largely the result of excess body weight and physical inactivity. A diagnosis of T2D was defined as symptoms such as polyuria or polydipsia, plus:[25 26]

- a random blood plasma glucose concentration ≥ 11.1 mmol/l or;
- a fasting plasma glucose concentration ≥ 7.0 mmol/l (whole blood ≥ 6.1 mmol/l) or;
- two-hour plasma glucose concentration ≥ 11.1 mmol/l two hours after 75g anhydrous glucose in an oral glucose tolerance test (OGTT) or;
- glycated haemoglobin (HbA1c) 6.5% or more (48 mmol/mol and above)

Relevant international classification of disease (ICD) codes used to define T2D will also be acceptable.

2) The World Health Organization (WHO) definition of body mass index (BMI) of 30 or above must have been used to define obesity.[27] BMI if calculated from self-reported height and weight or taken from medical records or measured at baseline will be acceptable.

3) Dyslipidaemia was classified as serum total cholesterol (TC), low density lipoprotein cholesterol (LDL-C), triglycerides, apolipoprotein B, or lipoprotein(a) concentrations above the 90th percentile, or high density lipoprotein cholesterol (HDL-C) or apolipoprotein concentrations below the 10th percentile for the general population.[28] Relevant ICD-codes used to define dyslipidaemia will also be acceptable.

4) The World Health Organization (WHO) definition of hypertension as:[29]

- a systolic blood pressure reading of ≥140 mmHg; and/or

- a diastolic blood pressure readings on both days of ≥90 mmHg, must have been used.

Relevant ICD-codes used to define hypertension will also be acceptable.

Outcome definitions:

All diagnoses of incident head and neck squamous cell carcinoma included in this study should have been confirmed by histology by a trained pathologist. Acceptable cancer diagnosis may be reported using ICD codes.[30] Cancer cases for the disease of interest have the following ICD-10 codes: oral cavity (C02.0-C02.9, C03.0-C03.9, C04.0-C04.9, C05.0-C06.9) oropharynx (C01.9, C02.4, C09.0-C10.9), hypopharynx (C13.0-C13.9), overlapping (C14 and combination of other sites) and 25 cases with unknown ICD code (other). Older versions of ICD code e.g., ICD-8 and 9 were also accepted. With respect to HPV diagnosis, only high risk subtypes HPV16, 18, 31, and 33 will be included. These are should be detected using HPV-DNA via polymerase chain reaction (PCR) and/or *in situ* hybridization (ISH) examination, usually used in combination with immunohistochemistry for *p16*.

Data analysis and synthesis

The analysis and synthesis process will be performed in two phases. The first phase consists of estimating the effect of each metabolic disorder (T2D, obesity, dyslipidemia and hypertension) on incident HNSCC separately for each included study. Effects should be reported as hazard ratios (HR), standardised incidence ratio (SIR), odds (OR) or risk ratios (RR), with 95% confidence intervals or in a format that will enable us to calculate these.

The second phase will focus on estimating the combined effect across studies using meta-analysis, using *metan* software in Stata (*Stata Statistical Software: Release 16*. College Station, TX: StataCorp LLC). Summary effects with their corresponding 95% CIs will be derived with the method of DerSimonian and Laird.[31] This will be performed separately for each metabolic disorder. The analysis will yield information about the heterogeneity of effects across studies, using Cochran's Q test and Higgins' I^2 statistic. Due to the heterogeneity in study design, exposure definition and outcome reporting in the included studies, meta-analysis may not be reasonable. If appropriate, to further explore the sources of heterogeneity if present, meta-regression analyses will be performed according to age, geographic location, year of publication, methods of exposure assessment, risk of bias score, length of follow-up, the number of cases and adjustment for confounding factors including smoking and alcohol use. Small study effects, which may reflect publication bias, study quality or other sources of heterogeneity, will be assessed using funnel plots and Egger's regression asymmetry tests.

Subgroup analyses

Where possible, we will stratify by oral, oropharyngeal, hypopharyngeal and laryngeal subsite and by HPV-status as post-hoc analyses, to determine if effects seen are specific to a particular anatomical region or associated with specific tumour types.

Risk of bias in studies

Two authors will independently extract the relevant risk factor information during data extraction using a preliminary version of the Risk of Bias in Non-randomised Studies – of Exposures (ROBINS-E) tool (https://www.bristol.ac.uk/population-health-

For peer review only - http://bmjopen.bmj.com/site/about/guidelines.xhtml

sciences/centres/cresyda/barr/riskofbias/robins-e/).[32] The ROBINS-E tool aims to assess the result of interest from an observational study for risk of bias, where that study is designed to assess the effect of exposure on outcome.[32] Disagreements will be recorded and discussed with a third author for resolution. This assessment will be used in evaluating the strength of evidence from the included studies.

Amendments

Any amendments to the initial protocol will be included in an update to the PROSPERO record, including information on amendment type, reasoning and a timestamp. Deviation from this protocol will be described in the full systematic review paper.

Dissemination

This systematic review will be published in a relevant peer-reviewed journal and findings from this work may be presented at scientific meetings (both poster and oral presentations).

Ethical approval

No ethical approval is required for the conducting of this systematic review.

Acknowledgements

None to declare.

References

1. Cancer Research UK (CRUK). Head and neck cancer statistics 2019 [cited 2019 11/04/2019]. Available from: https://www.cancerresearchuk.org/health-professional/cancer-statistics/statistics-by-cancer-type/head-and-neck-cancers#heading-Two accessed 13/09/2021 2019.

2. Thomas SJ, Penfold CM, Waylen A, et al. The changing aetiology of head and neck squamous cell cancer: A tale of three cancers? *Clin Otolaryngol* 2018;43(4):999-1003. doi: 10.1111/coa.13144

3. Office for National Statistics. Data for 1974 onwards: Office for National Statistics. Adult Smoking habits in Great Britain. 2020 [Available from: https://www.ons.gov.uk/peoplepopulationandcommunity/healthandsocialcare/drugusealcoholandsmoking/datasets/adultsmokinghabitsingreatbritain. accessed 01/03/2021 2020.

4. Office for National Statistics. Adult drinking habits in Great Britain 2017 [Available from: https://www.ons.gov.uk/peoplepopulationandcommunity/healthandsocialcare/drugusealcoholandsmoking/datasets/adultdrinkinghabits accessed 01/03/2021 2021.

5. Wotton CJ, Yeates DG, Goldacre MJ. Cancer in patients admitted to hospital with diabetes mellitus aged 30 years and over: record linkage studies. *Diabetologia* 2011;54(3):527-34. doi: 10.1007/s00125-010-1987-2 [published Online First: 2010/12/01]

6. Stott-Miller M, Chen C, Chuang SC, et al. History of diabetes and risk of head and neck cancer: a pooled analysis from the international head and neck cancer epidemiology

For peer review only - http://bmjopen.bmj.com/site/about/guidelines.xhtml

consortium. *Cancer Epidemiol Biomarkers Prev* 2012;21(2):294-304. doi: 10.1158/1055-9965.EPI-11-0590 [published Online First: 2011/12/07]

7. Vilaseca I, Fuster G, Avilés-Jurado FX. The impact of diabetes in head and neck cancer. *Curr Opin Otolaryngol Head Neck Surg* 2020;28(2):107-11. doi: 10.1097/moo.0000000000000606 [published Online First: 2020/02/06]

8. Rego DF, Elias ST, Amato AA, et al. Anti-tumor effects of metformin on head and neck carcinoma cell lines: A systematic review. *Oncol Lett* 2017;13(2):554-66. doi: 10.3892/ol.2016.5526 [published Online First: 2017/03/31]

9. Hanahan D, Weinberg RA. Hallmarks of Cancer: The Next Generation. *Cell* 2011;144(5):646-74. doi: 10.1016/j.cell.2011.02.013

10. Koivula RW, Heggie A, Barnett A, et al. Discovery of biomarkers for glycaemic deterioration before and after the onset of type 2 diabetes: rationale and design of the epidemiological studies within the IMI DIRECT Consortium. *Diabetologia* 2014;57(6):1132-42. doi: 10.1007/s00125-014-3216-x [published Online First: 2014/04/04]

11. Ogurtsova K, da Rocha Fernandes JD, Huang Y, et al. IDF Diabetes Atlas: Global estimates for the prevalence of diabetes for 2015 and 2040. *Diabetes Res Clin Pract* 2017;128:40-50. doi: 10.1016/j.diabres.2017.03.024 [published Online First: 2017/04/25]

12. Wolf AM, Colditz GA. Current estimates of the economic cost of obesity in the United States. *Obes Res* 1998;6(2):97-106. doi: DOI 10.1002/j.1550-8528.1998.tb00322.x

For peer review only - http://bmjopen.bmj.com/site/about/guidelines.xhtml

13. Dombrowski SU, Knittle K, Avenell A, et al. Long term maintenance of weight loss with non-surgical interventions in obese adults: systematic review and meta-analyses of randomised controlled trials. *Bmj-Brit Med J* 2014;348 doi: ARTN g2646 10.1136/bmj.g2646

14. Gray LJ, Cooper N, Dunkley A, et al. A systematic review and mixed treatment comparison of pharmacological interventions for the treatment of obesity. *Obes Rev* 2012;13(6):483-98. doi: 10.1111/j.1467-789X.2011.00981.x

15. Yanovski SZ, Yanovski JA. Long-term Drug Treatment for Obesity A Systematic and Clinical Review. *Jama-J Am Med Assoc* 2014;311(1):74-86. doi: 10.1001/jama.2013.281361

16. Corbin LJ, Timpson NJ. Body Mass Index: Has Epidemiology Started to Break Down Causal Contributions to Health and Disease? *Obesity* 2016;24(8):1630-38. doi: 10.1002/oby.21554

17. Maasland DH, van den Brandt PA, Kremer B, et al. Body mass index and risk of subtypes of head-neck cancer: the Netherlands Cohort Study. *Sci Rep* 2015;5:17744. doi: 10.1038/srep17744 [published Online First: 2015/12/05]

18. Gaudet MM, Kitahara CM, Newton CC, et al. Anthropometry and head and neck cancer:a pooled analysis of cohort data. *Int J Epidemiol* 2015;44(2):673-81. doi: 10.1093/ije/dyv059 [published Online First: 2015/06/08]

19. Stott-Miller M, Chen C, Schwartz SM. Type II diabetes and metabolic syndrome in relation to head and neck squamous cell carcinoma risk: a SEER-Medicare database

For peer review only - http://bmjopen.bmj.com/site/about/guidelines.xhtml

study. *Cancer Epidemiol* 2013;37(4):428-33. doi: 10.1016/j.canep.2013.03.006 [published Online First: 2013/04/09]

20. Kao LT, Hung SH, Kao PF, et al. Inverse association between statin use and head and neck cancer: Population-based case-control study in Han population. *Head Neck-J Sci Spec* 2019;41(5):1193-98. doi: 10.1002/hed.25501

21. Yen YC, Lin C, Lin SW, et al. Effect of metformin on the incidence of head and neck cancer in diabetics. *Head Neck* 2015;37(9):1268-73. doi: 10.1002/hed.23743 [published Online First: 2014/05/08]

22. Ramteke P, Deb A, Shepal V, et al. Hyperglycemia Associated Metabolic and Molecular Alterations in Cancer Risk, Progression, Treatment, and Mortality. *Cancers (Basel)* 2019;11(9) doi: 10.3390/cancers11091402 [published Online First: 2019/09/25]

23. Covidence. Covidence - Better systematic review management 2021 [Available from: https://www.covidence.org/ accessed 13/09/2021 2021.

24. Liberati A, Altman DG, Tetzlaff J, et al. The PRISMA statement for reporting systematic reviews and meta-analyses of studies that evaluate healthcare interventions: explanation and elaboration. *BMJ* 2009;339:b2700. doi: 10.1136/bmj.b2700

25. World Health Organization. Definition and Diagnosis of Diabetes Mellitus and Intermediate Hyperglycaemia 2006 [Available from: https://www.diabetes.org.uk/resources-s3/2017-09/Definition_and_diagnosis1111.pdf accessed 01/03/2021 2021.

26. World Health Organization. Use of Glycated Haemoglobin (HbA1c) in the Diagnosis of Diabetes Mellitus 2011 [Available from: https://www.diabetes.org.uk/resources-s3/2017-09/Definition_and_diagnosis1111.pdf accessed 01/03/2021 2021.

27. World Health Organization. Obesity and overweight 2021 [Available from: https://www.who.int/news-room/fact-sheets/detail/obesity-and-overweight accessed 01/03/2021 2021.

28. British Medical Journal. Hypercholesterolemia 2019 [Available from: https://bestpractice.bmj.com/topics/en-us/170 accessed 28.03.2021 2021.

29. Organization WH. Hypertension 2021 [Available from: https://www.who.int/news-room/fact-sheets/detail/hypertension accessed 13/09/2021 2021.

30. Organization WH. International Statistical Classification of Diseases and Related Health Problems (ICD) 2021 [accessed 13/09/2021 2021.

31. DerSimonian R, Laird N. Meta-analysis in clinical trials. *Control Clin Trials* 1986;7(3):177-88. doi: 10.1016/0197-2456(86)90046-2 [published Online First: 1986/09/01]

32. Bristol Uo. The ROBINS-E tool (Risk Of Bias In Non-randomized Studies - of Exposures) 2021 [Available from: http://www.bristol.ac.uk/population-health-sciences/centres/cresyda/barr/riskofbias/robins-e/ accessed 13/09/2021 2021.

Authors' contributions

AG and CR jointly contributed to the development of the protocol and the drafting, writing and editing of this manuscript. FS contributed to the development of this study and editing

of the manuscript. JH contributed to the development of the search strategy. BM, EG, EV, RR and JPTH contributed to the development of this study. MG was responsible for conceptualising this study, drafting and editing this manuscript. All authors contributed to the development of the search strategy. All authors have approved and contributed to the final written manuscript.

Funding statement

A.G. is a National Institute for Health Research (NIHR) academic clinical fellow (no grant number). F.S. was supported by a Cancer Research UK (C18281/A29019) programme grant (the Integrative Cancer Epidemiology Programme). E.E.V is supported by Diabetes UK (17/0005587). E.E.V is also supported by the World Cancer Research Fund (WCRF UK), as part of the World Cancer Research Fund International grant programme (IIG_2019_2009). R.C.R. is a de Pass VC research fellow at the University of Bristol (no grant number). J.P.T.H supported by the NIHR Biomedical Research Centre at University Hospitals Bristol and Weston NHS Foundation Trust and the University of Bristol (no grant number), the NIHR Applied Research Collaboration West at University Hospitals Bristol and Weston NHS Foundation Trust (no grant number) and is a National Institute for Health Research (NIHR) Senior Investigator (no grant number). M.G. is currently supported by a Wellcome Trust GW4-Clinical Academic Training PhD Fellowship. This research was funded in part, by the Wellcome Trust [Grant number 220530/Z/20/Z]. For the purpose of Open Access, the author has applied a CC BY public copyright licence to any Author Accepted Manuscript version arising from this submission. The views expressed in this publication are those of the author(s) and not necessarily those of Wellcome, NIHR, the NHS or Department of Health.

Competing interests statement

None declared

Patient and Public Involvement

Patients and/or the public were not involved in the development, conduct or proposed dissemination of this systematic review protocol.

Word Count*:* 1,956 (excluding abstract)

Supplementary Table 1. Search strategy.

Supplementary Table 2. Modified search strategy.

Supplementary Table 3. Data extraction table. Note separate table for each exposure (Obesity, Dyslipidaemia, Hypertension, Type 2 diabetes).

Supplementary Checklist 1. PRISMA-P checklist.

For peer review only - http://bmjopen.bmj.com/site/about/guidelines.xhtml

Supplementary Table 1. Search strategy

1
exp "HEAD AND NECK NEOPLASMS"/
2
("head and neck cancer" or "oral cancer").ti,ab.
3
((lip or lips or mouth or gums or palatal or palate or gingiva* or buccal or cheek* or tongue or lingual or glosso* or laryn* or "vocal chord*" or "vocal cord*" or "vocal fold*" or subglottic or supraglottic or hypopharyn* or hypo-pharyn* or epiglottis or arytenoids or aryepiglottic or throat or oropharyn* or oro-pharyn* or tonsil* or pharyn* or otolaryn* or jaw or mandib* or maxill*) adj3 (cancer* or carcinoma* or neoplasm* or tumour* or tumor* or squamous or lymphoma* or HPV or "human papillomavirus")).ti,ab.
4
1 or 2 or 3
5
exp DYSLIPIDEMIAS/
6
(dyslipidaemia or dyslipidemia or lipolysis or hypercholesterolemia or hypercholesterolaemia or "low density lipoprotein" or “apolipoprotein” or cholesterol or LDL or triglycerides or statin* or ezetimibe or "HMG-CoA" or "reductase inhibitor*" or "HMGCR" or "hydroxymethylglutaryl coa reductases" or "NPC1L1" or "PCSK9" or "CETP" or "LDLR" or atorvastatin or fluvastatin or lovastatin or pitavastatin or pravastatin or rosuvastatin or simvastatin or (lipid* adj2 (metaboli* or medicat* or drug* or lower* or reduc* or decreas*))).ti,ab.
7
5 or 6
8
exp DIABETES MELLITUS/
9
(Diabet* or insulin or "insulin resistance" or hyperglycaemia or hypoglycaemia or hyperglycemia or hypoglycemia or hypertension or metformin or glucagon or "hypoglycaemic agent*" or HbA1 or HbA1c or (glucose adj2 (impair* or tolerance or intolerance)) or IGT or glycogenolysis or gluconeogenesis or GDF?15 or gliflozins or SGLT2 or sulphonylurea* or meglitinide* or thiazolidinediones or "DPP-4 inhibitor*" or "GLP-1 receptor agonist*").ti,ab.
10
8 or 9
11
exp METABOLIC SYNDROME/
12
(metabolic syndrome or "dyresgulated metaboli*" or obesity or obes* or "abdominal obes*" or (fat or fatty or fats) or "body weight" or adipos* or (weight adj2 gain*) or overeat* or bariatric or (over adj2 eat) or "body mass index*" or BMI).ti,ab.
13
11 or 12
14
7 or 10 or 13
15
4 and 14
16
exp RISK/
17
(risk* or inciden* or prevalen*).ti,ab.
18
16 or 17
19
15 and 18

For peer review only - http://bmjopen.bmj.com/site/about/guidelines.xhtml

Supplementary Table 2. Modified search strategy.

1

HEAD AND NECK CANCER

2

AND

3

CHOLESTEROL

4

OR

5

DIABETES

6

OR

7

METABOLIC SYNDROME

8

OR

9

OBESITY

For peer review only

Supplementary Table 3. Data extraction table. Note separate table for each exposure (Obesity, Dyslipidaemia, Hypertension, Type 2 diabetes).

Obesity

Study ID	First Author	Year	Study design	Sample size	Age in years (mean)	Age in years (SD)	Age in years (median)	Age in years (IQR)	Age in years (range)
Other measures of age	Sex (% of female)	Sex (% of male)	White	Black	Hispanic	Other	Ethnicity	Country	Population setting
Method of exposure diagnosis	Exposure (units)	Treatment taken for exposure	Method of outcome assessment	HPV status	Adjusted covariates	Outcome (subsite)	no. with obesity and outcome	Reported Statistic	Effect size
95% CI	Follow-up in years (mean)	Follow-up in years (SD)	Follow-up in years (median)	Follow-up in years (IQR)	Follow-up in years (range)	Other measures of follow-up in years			

Dyslipidaemia

Study ID	First Author	Year	Study design	Sample size	Age in years (mean)	Age in years (SD)	Age in years (median)	Age in years (IQR)	Age in years (range)
Other measures of age	Sex (% of female)	Sex (% of male)	White	Black	Hispanic	Other	Ethnicity	Country	Population setting
Exposure (units)	Method of exposure diagnosis	Treatment taken for exposure	Method of outcome assessment	HPV status	Adjusted covariates	Outcome (subsite)	no. with dyslipidaemia and outcome	Reported Statistic	Effect size
95% CI	Follow-up in years (mean)	Follow-up in years (SD)	Follow-up in years (median)	Follow-up in years (IQR)	Follow-up in years (range)	Other measures of follow-up in years			

Hypertension

Study ID	First Author	Year	Study design	Sample size	Age in years (mean)	Age in years (SD)	Age in years (median)	Age in years (IQR)	Age in years (range)
Other measures of age	Sex (% of female)	Sex (% of male)	White	Black	Hispanic	Other	Ethnicity	Country	Population setting
Exposure (units)	Method of exposure diagnosis	Treatment taken for exposure	Method of outcome assessment	HPV status	Adjusted covariates	Outcome (subsite)	no. with hypertension and outcome	Reported Statistic	Effect size
95% CI	Follow-up in years (mean)	Follow-up in years (SD)	Follow-up in years (median)	Follow-up in years (IQR)	Follow-up in years (range)	Other measures of follow-up in years			

For peer review only

Type 2 diabetes

Study ID	First Author	Year	Study design	Sample size	Age in years (mean)	Age in years (SD)	Age in years (median)	Age in years (IQR)	Age in years (range)
**Other measures of age**	**Sex (% of female)**	**Sex (% of male)**	**White**	**Black**	**Hispanic**	**Other**	**Ethnicity**	**Country**	**Population setting**
**Exposure (units)**	**Method of exposure diagnosis**	**Treatment taken for exposure**	**Method of outcome assessment**	**HPV status**	**Adjusted covariates**	**Outcome (subsite)**	**no. with type 2 diabetes and outcome**	**Reported Statistic**	**Effect size**
**95% CI**	**Follow-up in years (mean)**	**Follow-up in years (SD)**	**Follow-up in years (median)**	**Follow-up in years (IQR)**	**Follow-up in years (range)**	**Other measures of follow-up in years**			

For peer review only

Supplementary Checklist 1. PRISMA-P checklist.

Reporting checklist for protocol of a systematic review and meta analysis.

Instructions to authors

Complete this checklist by entering the page numbers from your manuscript where readers will find each of the items listed below.

Your article may not currently address all the items on the checklist. Please modify your text to include the missing information. If you are certain that an item does not apply, please write "n/a" and provide a short explanation.

Upload your completed checklist as an extra file when you submit to a journal.

In your methods section, say that you used the PRISMA-Preporting guidelines, and cite them as:

Moher D, Shamseer L, Clarke M, Ghersi D, Liberati A, Petticrew M, Shekelle P, Stewart LA. Preferred Reporting Items for Systematic Review and Meta-Analysis Protocols (PRISMA-P) 2015 statement. Syst Rev. 2015;4(1):1.

		Reporting Item	Page Number
**Title**			
Identification	#1a	Identify the report as a protocol of a systematic review	1
Update	#1b	If the protocol is for an update of a previous systematic review, identify as such	N/A
**Registration**			
	#2	If registered, provide the name of the registry (such as PROSPERO) and registration number	8
**Authors**			
Contact	#3a	Provide name, institutional affiliation, e-mail address of all protocol authors; provide physical mailing address of corresponding author	1
Contribution	#3b	Describe contributions of protocol authors and identify the guarantor of the review	18-19
**Amendments**			
	#4	If the protocol represents an amendment of a previously completed or published protocol, identify as such and list changes; otherwise, state plan for documenting important protocol amendments	N/A

For peer review only

**Support**			
Sources	#5a	Indicate sources of financial or other support for the review	19
Sponsor	#5b	Provide name for the review funder and / or sponsor	19
Role of sponsor or funder	#5c	Describe roles of funder(s), sponsor(s), and / or institution(s), if any, in developing the protocol	19
**Introduction**			
Rationale	#6	Describe the rationale for the review in the context of what is already known	6
Objectives	#7	Provide an explicit statement of the question(s) the review will address with reference to participants, interventions, comparators, and outcomes (PICO)	7
**Methods**			
Eligibility criteria	#8	Specify the study characteristics (such as PICO, study design, setting, time frame) and report characteristics (such as years considered, language, publication status) to be used as criteria for eligibility for the review	7
Information sources	#9	Describe all intended information sources (such as electronic databases, contact with study authors, trial registers or other grey literature sources) with planned dates of coverage	8-9
Search strategy	#10	Present draft of search strategy to be used for at least one electronic database, including planned limits, such that it could be repeated	20
Study records - data management	#11a	Describe the mechanism(s) that will be used to manage records and data throughout the review	9
Study records - selection process	#11b	State the process that will be used for selecting studies (such as two independent reviewers) through each phase of the review (that is, screening, eligibility and inclusion in meta-analysis)	9
Study records - data collection process	#11c	Describe planned method of extracting data from reports (such as piloting forms, done independently, in duplicate), any processes for obtaining and confirming data from investigators	9
Data items	#12	List and define all variables for which data will be sought (such as PICO items, funding sources), any pre-planned data assumptions and simplifications	20
Outcomes and prioritization	#13	List and define all outcomes for which data will be sought, including prioritization of main and additional outcomes, with rationale	11
Risk of bias in individual studies	#14	Describe anticipated methods for assessing risk of bias of individual studies, including whether this will be done at the	12-13

For peer review only

		outcome or study level, or both; state how this information will be used in data synthesis	
Data synthesis	#15a	Describe criteria under which study data will be quantitatively synthesised	11-12
Data synthesis	#15b	If data are appropriate for quantitative synthesis, describe planned summary measures, methods of handling data and methods of combining data from studies, including any planned exploration of consistency (such as I2, Kendall's τ)	11-12
Data synthesis	#15c	Describe any proposed additional analyses (such as sensitivity or subgroup analyses, meta-regression)	11-12
Data synthesis	#15d	If quantitative synthesis is not appropriate, describe the type of summary planned	N/A
Meta-bias(es)	#16	Specify any planned assessment of meta-bias(es) (such as publication bias across studies, selective reporting within studies)	11-12
Confidence in cumulative evidence	#17	Describe how the strength of the body of evidence will be assessed (such as GRADE)	11-12

The PRISMA-P elaboration and explanation paper is distributed under the terms of the Creative Commons Attribution License CC-BY. This checklist was completed on 13. September 2021 using https://www.goodreports.org/, a tool made by the EQUATOR Network in collaboration with Penelope.ai

For peer review only
